# Respiratory Syncytial Virus in Hematopoietic Cell Transplant Recipients: Clinical and Humoral Risk Factors for Infection

**DOI:** 10.1093/ofid/ofag005

**Published:** 2026-01-20

**Authors:** Sara Pernikoff, Annelie Clurman, Melanie Rötepohl, Nina Galanter, Madeleine Bibby, Evelyn Harris, Terry Stevens-Ayers, Hu Xie, Masumi Ueda Oshima, Guang-Shing Cheng, Janet A Englund, Michael J Boeckh, Jim Boonyaratanakornkit

**Affiliations:** Vaccine and Infectious Disease Division, Fred Hutchinson Cancer Center, Seattle, Washington, USA; Vaccine and Infectious Disease Division, Fred Hutchinson Cancer Center, Seattle, Washington, USA; School of Medicine, University of Washington, Seattle, Washington, USA; Vaccine and Infectious Disease Division, Fred Hutchinson Cancer Center, Seattle, Washington, USA; Department of Biostatistics, University of Washington, Seattle, Washington, USA; Vaccine and Infectious Disease Division, Fred Hutchinson Cancer Center, Seattle, Washington, USA; Vaccine and Infectious Disease Division, Fred Hutchinson Cancer Center, Seattle, Washington, USA; Vaccine and Infectious Disease Division, Fred Hutchinson Cancer Center, Seattle, Washington, USA; Vaccine and Infectious Disease Division, Fred Hutchinson Cancer Center, Seattle, Washington, USA; Department of Medicine, University of Washington, Seattle, Washington, USA; Clinical Research Division, Fred Hutchinson Cancer Center, Seattle, Washington, USA; Department of Medicine, University of Washington, Seattle, Washington, USA; Clinical Research Division, Fred Hutchinson Cancer Center, Seattle, Washington, USA; Seattle Children's Research Institute and Department of Pediatrics, University of Washington, Seattle, Washington, USA; Vaccine and Infectious Disease Division, Fred Hutchinson Cancer Center, Seattle, Washington, USA; Department of Medicine, University of Washington, Seattle, Washington, USA; Vaccine and Infectious Disease Division, Fred Hutchinson Cancer Center, Seattle, Washington, USA; Department of Medicine, University of Washington, Seattle, Washington, USA

**Keywords:** antibodies, graft-versus-host disease, hematopoietic cell transplant, immunosuppression, respiratory syncytial virus, neutralization, risk factors

## Abstract

**Background:**

Respiratory syncytial virus (RSV) frequently causes upper respiratory tract infections, lung disease, and mortality in hematopoietic cell transplant (HCT) recipients. Currently, little is understood about what clinical and immunologic factors increase a patient's risk of infection or are protective against infection in immunocompromised populations.

**Methods:**

This study analyzed clinical and serologic data from a cohort of HCT recipients followed longitudinally with weekly blood draws and PCR surveillance for respiratory viruses to gain insight into clinical and antibody-based risk factors for RSV infection post-transplant. Serum was analyzed by a plaque reduction neutralization assay to determine neutralizing antibody titers to RSV.

**Results:**

Sixteen of 471 HCT recipients tested positive for RSV within the first 100 days post-transplant. A multivariate analysis of clinical factors revealed that prophylaxis with sirolimus for graft-versus-host disease (GVHD) was significantly correlated with increased risk of RSV infection. Moreover, higher levels of neutralizing antibody to RSV were associated with reduced risk for RSV infection, in a time-varying analysis.

**Conclusions:**

GVHD prophylaxis with sirolimus and low serum neutralizing antibody titers were correlated with increased risk of RSV infection in the early post-transplant period. These results support the role of developing and implementing strategies that boost neutralizing antibody levels to prevent RSV infections in HCT recipients.

## Teaser

Two major findings were found from a prospective cohort of hematopoietic cell transplant recipients. (1) There is a significant correlation between immunosuppression for graft-versus-host disease and increased risk for RSV infection post-transplant. (2) There is a significant correlation between lower antibody levels and increased risk for RSV infection post-transplant.

Respiratory viral infections are a significant cause of mortality, morbidity, and high health care costs for hematopoietic cell transplant (HCT) recipients [1]. In the early period after HCT, recipients have incomplete humoral immune reconstitution [2–4], increasing their susceptibility to respiratory complications, which occur in 45%-60% of HCT recipients [5–7]. Respiratory syncytial virus (RSV) is a frequent cause of respiratory viral infection after HCT [1, 8] and can lead to severe progressive disease in HCT recipients, with a mortality rate of 20%-40% despite currently available treatment interventions [9, 10].

Vaccination and/or passive prophylaxis with monoclonal antibodies (mAbs) are potential strategies to prevent viral infection in the URT or progression to the LRT in HCT recipients [4, 11, 12]. For example, the long acting mAb, nirsevimab, targets a conformational epitope on the F protein and was recently approved for prophylaxis of RSV in infants [13, 14]. Another long-acting mAb, clesrovimab, targets a different antigenic site present on F and has similarly shown efficacy in reducing RSV disease and hospitalization in infants [15]. Three vaccines have also been approved for the prevention of RSV infection during pregnancy and/or in adults 60 years of age and over [16–18], with 1 vaccine licensed for high risk adults based on immunogenicity studies [19]. Unfortunately, there is still a large unmet need for the prevention of RSV in immunocompromised patients [11].

The impact of serum neutralizing antibodies on protection against RSV infection in healthy adults is not clear, with even less known about correlates of protection in immunocompromised individuals [20, 21]. Several lines of evidence suggest that immunologic factors confer protection against infection with respiratory viruses after HCT. For example, HCT recipients have a higher risk of developing pneumonia from RSV in the pre-engraftment than in the post-engraftment period [22]. Furthermore, allogeneic transplant recipients are almost 10-times more likely than autologous HCT recipients to acquire a viral infection in the upper respiratory tract leading to symptoms [23].

The aim of this study was to identify clinical and humoral immune factors associated with risk of RSV infection up to 100-days post-transplant in a longitudinal cohort of HCT recipients. Serum neutralization assays were performed to determine if lower serum RSV neutralizing antibody levels in HCT recipients were associated with increased risk of RSV infection post-transplant. These results could guide the development and implementation of therapeutic and prophylactic modalities for RSV in HCT recipients.

## METHODS

### Cohort

**Figure 1.**
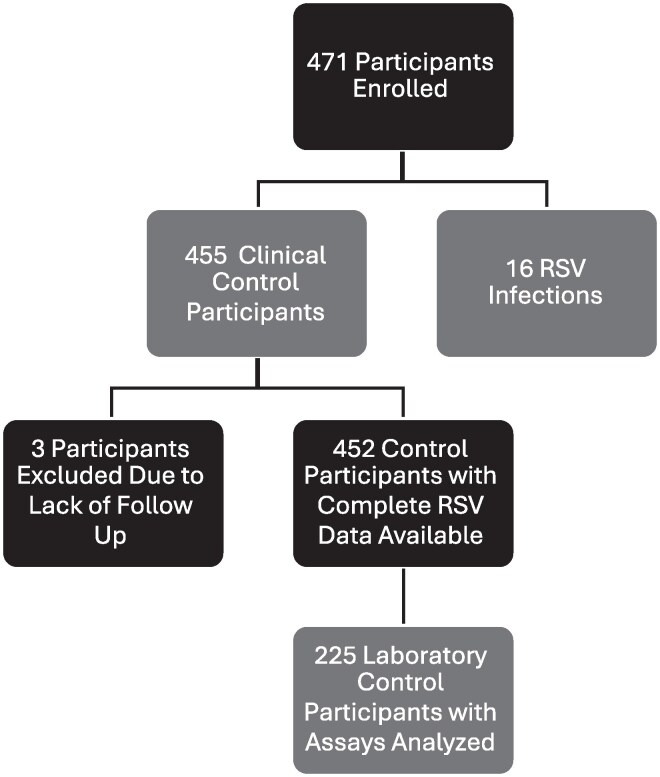
Study schema. HCT recipients were enrolled and followed longitudinally with weekly sampling of serum and nasal washes for PCR detection of respiratory viruses. Patients with RSV detected by PCR (RSV group) were analyzed in comparison to a control group without RSV detected by PCR (control group).

This study utilized clinical data and patient samples from a prospective cohort of 471 allogeneic HCT recipients at Fred Hutchinson Cancer Center transplanted between 2005 and 2010 [24, 25]. Patients were enrolled prior to transplant and participated in weekly symptom and exposure surveys, as well as nasal washes and serum collection for up to 1-year post-transplant. RSV was detected in nasal washes using a laboratory developed multiplex PCR as previously described [26]. Serologic laboratory analyses were conducted on all participants with PCR-positive detection of RSV (excluding 1 individual due to lack of follow-up) and 225 (approximately 50%) of control participants who had serum collected up to day 100 but no detection of RSV (Figure 1). The control group of 225 patients was chosen based on sample availability for the pre-transplant/peri-transplant and post-transplant time points. In addition, we prespecified analyzing approximately half of the overall control group, given the limited number of cases (*N* = 16) and feasibility constraints of performing neutralization assays on all controls. There were no substantive differences in demographic variables between the sampled control cohort versus the overall control cohort, except, as expected, the sampled control cohort had longer follow-up due to the sampling schema (Supplementary Table S1). Observations were included in the laboratory analysis based on whether individuals were followed up through the initial 2 time points. These time points consisted of a pre-transplant/peri-transplant time point between 18 days pre-transplant and 7 days post-transplant, and a second post-transplant time point between 8 and 51 days post-transplant [26].

### Neutralization Assays

Vero Cells (ATCC CCL-81) and Hep2 cells (ATCC CCL-23) were cultured in DMEM (Thermo Fisher, cat#11965-118) supplemented with 10% fetal bovine serum (Cytiva, cat#SH30071.03), penicillin (100 U/mL) and streptomycin (100 µg/mL) (Gibco, cat#15140-122). Recombinant virus was modified to express GFP from RSV strain A2 (GenBank accession number KT992094) and has been previously described [27, 28]. RSV-GFP was cultured on Hep2 cells and titers were determined by infecting Vero cell monolayers with serial 10-fold dilutions in 24-well plates. The infected cells were overlayed with DMEM containing 0.8% methylcellulose (Sigma Aldrich, cat#M0387). Five days post-infection, fluorescent plaques were detected with a Typhoon Trio imager (Cytiva) and counted in ImageJ [29].

To determine serum neutralization titers, Vero cells were seeded in flat-bottom 96-well plates, 48 hours before each assay. Serum was diluted to 1:100 and 1:1000 for serum in DMEM supplemented with penicillin (100 U/mL), streptomycin (100 µg/mL), and amphotericin B (2.5 µg/mL) (Gibco, cat#15290-018). The WHO International Standard, first International Standard for Antiserum to Respiratory Syncytial Virus (NIBSC code: 16/284) was used as a control to establish standard curves for neutralization assays. The standard curve was set up using a 4-fold serial dilution of the WHO standard from 1:20 to 1:20,480. Fifty microliter of RSV-GFP (diluted to 6,000 plaque-forming units (pfu)/mL) was incubated with either 40 µL of each sample dilution or of each dilution of the WHO standard for 1 hour at 37°C. Vero cells were then incubated for 1 hour at 37°C with 50 µL of this mixture before being overlayed with 100 µL of DMEM containing 0.8% methylcellulose, penicillin (100 U/mL), and streptomycin (100 µg/mL). Five days post-infection, fluorescent plaques were detected with a Typhoon Trio imager and counted in ImageJ. All samples, the WHO standard, and negative controls were run in duplicate and averaged.

### Antibody Value Interpolation

Using GraphPad Prism software, the WHO standard was used to generate a standard curve, and antibody values were interpolated based on this curve using a sigmoidal, 4 parameter logistic equation for the log base 10 of the dilution factor. Serum samples with values outside of the standard curve were imputed at the next 4-fold dilution (Supplementary Table S2). As the serum samples included 2 dilutions, the dilution with interpolated value closest to the center of the standard curve on the log scale was used in the statistical analysis; if both dilutions were out of range at opposite ends of the standard curve, then the sample was not included in the analysis. This occurred for 1 sample from 1 participant.

### Statistical Analysis

R version 4.2.0 was used for all analyses. The initial time point was day of transplant, except in the post-transplant antibody analyses, and the event was RSV detection by PCR. Patients were censored 100 days after transplant or after their last observed PCR test, whichever occurred earlier. For the clinical correlates analyses, a univariate Cox proportional hazards model was used to estimate hazard ratios for each clinical variable. Post-baseline measures (e.g., blood cell counts) were adjusted in the model as time-varying covariates with the last value carried forward. All measures for which the *P* value was under 0.2 were considered for inclusion in a multivariate Cox proportional hazards model. For all models, the standard Wald *P* value was used. For each analysis, patients missing any of the relevant covariate values were excluded. For the analysis of serum neutralizing antibodies, inverse propensity of missingness weighting was used to account for the sampling scheme, with the bootstrap method (50,000 resamples with both estimation of the weights and the final model) used to calculate confidence intervals and significance levels. As samples were selected partially based on completeness of follow-up at the pre-transplant/peri-transplant time point (18 days before to 7 days after transplant) and the post-transplant time point (9 to 51 days after transplant), cytomegalovirus status at baseline and whether the transplant was myeloablative were used to model loss to follow-up.

The outcome considered for the primary analysis was first detected infection with RSV. Our cohort also included data for first detected infection with human metapneumovirus virus (HMPV), first detected infection with human parainfluenza virus type 3 (HPIV3), first detected infection with any of the 3 viruses (including RSV), virus-specific nasal neutralizing titers, virus-specific nasal IgA, and virus-specific serum neutralizing titers. RSV detection by PCR and RSV-specific serum neutralizing antibody titers were selected as the primary analysis because the other outcomes were judged as unreliable due to low event counts or not scientifically meaningful in the case of the combined virus outcome. Nasal antibody correlates were deemed exploratory and nasal wash data was unavailable to allow for correction of varying dilutions during the nasal wash procedure.

For the pre-transplant/peri-transplant analysis of antibody levels, univariate Cox proportional hazard models were used. For the post-transplant analysis, univariate Cox proportional hazard models were used with the initial time point being the day relative to transplant on which serum was collected for the antibody measurement. Patients for whom an RSV infection was detected prior to the antibody measure were removed from the analyses, meaning these results only apply to those who were not infected before the time of measurement. For the time-varying analysis, antibody measurements were included in the model as time-varying covariates with the last value carried forward until 28 days after the most recent sample, at which point individuals were considered out of follow-up until the next available antibody measurement. Since samples were obtained weekly rather than daily, antibody observations were not included if they occurred 7 or less days before infection with RSV to help ensure antibody observations in the time-varying analysis pre-dated infection.

In addition to RSV detection, we performed exploratory analyses on time to detected infection with HMPV, time to detected infection with HPIV3, and time to detected infection with any 1 of HMPV, RSV, or HPIV3 (Supplementary Table S3). No significant correlations were identified. We determined that the results for HMPV were unreliable due to an even lower event rate than RSV (*N* = 10). The combined virus detection outcome was explored to increase event counts, but serum neutralizing antibodies to 1 virus, for example RSV, are generally not protective against another virus, for example HPIV3. We also explored neutralization and IgA binding to fusion proteins stabilized in the prefusion conformation of RSV, HMPV, and HPIV3 in nasal wash specimens. No significant correlations were identified, although these analyses were limited due to the lack of correction for varying levels of dilution of the nasal wash samples. These exploratory analyses are summarized in Supplementary Table S3. We did not conduct multiple testing adjustments due to the exploratory nature of these analyses.

## RESULTS

### Study Population and Clinical Characteristics

A longitudinal prospective cohort of allogeneic HCT recipients was enrolled at Fred Hutch Cancer Center between 2005 and 2010. Of the 471 allogeneic HCT recipients enrolled, 468 underwent at least 1 weekly symptom and exposure survey for respiratory viral infections, nasal washes, and serum collection (Figure 1). The prospective weekly sampling of serum and nasal washes offered a powerful opportunity to evaluate humoral immunity prior to infection. The median length of follow-up was 186.5 days with an IQR of 93.8 to 363.0 days.

All nasal washes were tested for RSV by PCR. Sixteen (3.4%) out of 471 recipients tested positive within the first 100 days post-transplant. Of those that tested positive, the median time from transplant to detected infection was 37.5 days, with an IQR of 13.5 to 74.5 days. The 452 HCT recipients who never tested positive for RSV in the first 100 days post-transplant and were followed were included as a control group for the clinical analysis; approximately half (*N* = 225) were included as the control group for laboratory analysis.

The mean age of control participants who tested negative for RSV was 46 years, while patients that tested positive had a mean age of 53 years (Table 1). Participant ages ranged from 1 to 75 years, with 24 (5%) participants being 10 or younger and 120 (26%) being 60 or older. The distribution of underlying diseases for HCT is shown in Table 1. Only 3 participants received intravenous immunoglobulins (IVIG) within the first 100 days post-transplant, and all 3 were in the control group. Of the 16 patients with RSV detected in the first 100 days, 4 (25%) were hospitalized with RSV and 3 (19%) presented with involvement of the LRT (Supplementary Table S4). Only 1 participant acquired RSV during their admission for transplant. Four (25%) with RSV infection received ribavirin, and none of the patients experienced death associated with or attributed to RSV infection within 100 days post-transplant.

**Table 1.** Univariate Analysis of Baseline Clinical Variables and Their Associated Risk of RSV Infection

				Univariate Analysis		
Variable	Total (*n* = 471)	Control (*n* = 455)	RSV (*n* = 16)	Hazard Ratio	95% CI	*P* value
Days follow-up [mean (SD)]	215 (137)	214 (137)	243 (137)	NA	NA	NA
Recipient age [mean (SD, range)]	46 (18, 1-75)	46 (18,1-75)	53 (16,15-73)	1.03	(.99, 1.06)	.15
Recipient age 10 and younger	24 (5)	24 (5)	0 (0)	NA	NA	NA
Recipient age 60 and older	120 (26)	115 (25)	5 (31)	1.37	(.48, 3.95)	.56
Recipient Sex—male	299 (64)	289 (64)	10 (63)	0.96	(.35, 2.64)	.94
Diagnosis						
AML	164 (35)	159 (35)	5 (31)	Reference		…
ALL	58 (12)	56 (12)	2 (13)	1.19	(.23, 6.12)	.84
Chronic leukemia	45 (10)	43 (10)	2 (12)	1.38	(.27, 7.13)	.70
Lymphomas	51 (11)	49 (11)	2 (13)	1.27	(.25, 6.57)	.77
MDS	54 (12)	52 (11)	2 (13)	1.29	(.25, 6.64)	.76
Myeloma	34 (7)	32 (7)	2 (13)	1.88	(.37, 9.70)	.45
Other diseases	65 (14)	64 (14)	1 (6)	0.47	(.06, 4.03)	.49
Smoking status						
Never smoked	290 (62)	281 (62)	9 (56)	Reference		…
Formerly smoked	130 (28)	125 (28)	5 (31)	1.32	(.44, 3.94)	.62
Currently smokes	32 (7)	30 (7)	2 (13)	2.17	(.47, 10.04)	.32
Not reported^a^	19 (4)	19 (4)	0	Excluded		…
Transplant type						
Nonmyeloablative or reduced intensity	197 (42)	189 (42)	8 (50)	Reference		…
Myeloablative + no TBI	145 (31)	140 (31)	5 (31)	0.84	(.27, 2.56)	.75
Myeloablative + TBI	129 (27)	126 (28)	3 (19)	0.61	(.16, 2.30)	.46
Exposure to children						
No exposure	313 (67)	303 (67)	10 (63)	Reference		…
Exposure to children 0-10 years old	145 (31)	139 (31)	6 (38)	1.33	(.48, 3.66)	.58
Exposure to children under 5 years old	90 (19)	87 (19)	3 (19)	0.92	(.25, 3.32)	.90
Exposure to Children 5-10 years old	87 (18)	83 (18)	4 (25)	1.53	(.48, 4.86)	.47
Not reported^a^	13 (3)	13 (3)	0	Excluded		…
Season of transplant						
Fall	104 (22)	97 (21)	7 (44)	Reference		…
Spring	105 (22)	101 (22)	4 (25)	0.57	(.17, 1.94)	.37
Summer^a^	131 (28)	131 (29)	0	NA	NA	NA
Winter	131 (28)	126 (28)	5 (31)	0.57	(.18, 1.81)	.34
Year of transplant						
2006	96	93	3	Reference		…
2007	114	104	10	2.97	(.82, 10.78)	.10
2008	125	122	3	0.80	(.16, 3.96)	.78
2009	119	199	0	NA	NA	NA
2010	11	11	0	NA	NA	NA
Graft type						
Related/matched	157 (33)	150 (33)	7 (44)	Reference		…
Related/haploidentical^a^	22 (5)	22 (5)	0	NA	NA	NA
Related/mismatch	8 (2)	7 (2)	1 (6)	2.67	(.33, 21.74)	.36
Unrelated/cord^a^	45 (10)	45 (10)	0	NA	NA	NA
Unrelated/matched	212 (45)	205 (45)	7 (4)	0.74	(.26, 2.12)	.58
Unrelated/mismatch	27 (6)	26 (6)	1 (6)	0.85	(.10, 6.91)	.88
Graft source						
Bone marrow	83 (18)	81 (18)	2 (13)	Reference		…
Cord blood^a^	45 (10)	45 (10)	0	NA	NA	NA
Peripheral blood	343 (73)	329 (72)	14 (88)	1.66	(.38, 7.31)	.50
Blood counts^b^						
AMC < 100	NA	NA	NA	0.25	(.04, 1.4)	.11
ANC < 1000	NA	NA	NA	0.14	(.03, .70)	.02
ALC < 100	NA	NA	NA	0.24	(.03, 2.03)	.19
WBC (per 1,000)	NA	NA	NA	1.02	(.95, 1.08)	.63
GVHD prophylaxis medications^c^						
Post-transplant cyclophosphamide included^a^	25 (5)	25 (6)	0	NA	NA	NA
Rapamycin (sirolimus) included	18 (4)	14 (3)	4 (25)	8.85	(2.85, 27.43)	< .01
Calcineurin inhibitors included^a^	467 (99)	451 (99)	16 (100)	NA	NA	NA
Mycophenolate included	253 (54)	245 (54)	8 (50)	0.87	(.33, 2.32)	.78
Methotrexate included	212 (45)	204 (45)	8 (50)	1.21	(.45, 3.22)	.70
IVIG administration^d^	3 (<1)	3 (<1)	0 (0)	NA	NA	NA
Systemic Steroids^b^	NA	NA	NA	1.78	(.57, 5.54)	.32
CMV status						
D^−^/R^−^	157 (33)	151 (33)	6 (38)	Reference		…
D^−^/R^+^	163 (35)	160 (35)	3 (19)	0.46	(.12, 1.85)	.28
D^+^/R^−^	41 (9)	40 (9)	1 (6)	0.58	(.07, 4.83)	.62
D^+^/R^+^	110 (23)	104 (23)	6 (38)	1.41	(.45, 4.36)	.56

Abbreviations: AML, acute myeloid leukemia; ALL, acute lymphoblastic leukemia; MDS, myelodysplastic syndrome; TBI, total body irradiation; AMC, absolute monocyte count; ANC, absolute neutrophil count; ALC, absolute lymphocyte count; WBC, white blood cell count; GVHD, graft-versus-host disease; IVIG, intravenous immunoglobulin; CMV, cytomegalovirus; D/R, donor/recipient; NA, not applicable.

^a^Hazard ratios for categories in which no events occurred or all events occurred could not be estimated.

^b^Blood counts including ANC, AMC, ALC, and WBC as well as systemic steroid usage were calculated as time dependent variables. Steroid usage was documented through a weekly symptom survey asking if participants were taking systemic steroids within the prior week (yes/no).

^c^Some participants were on multiple medications. The reference group for each medication was all individuals that did not receive the medication listed.

^d^IVIG was administered within 100 days post-transplant.

### Clinical Risk Factors for RSV Acquisition Post-Transplant

**Figure 2.**
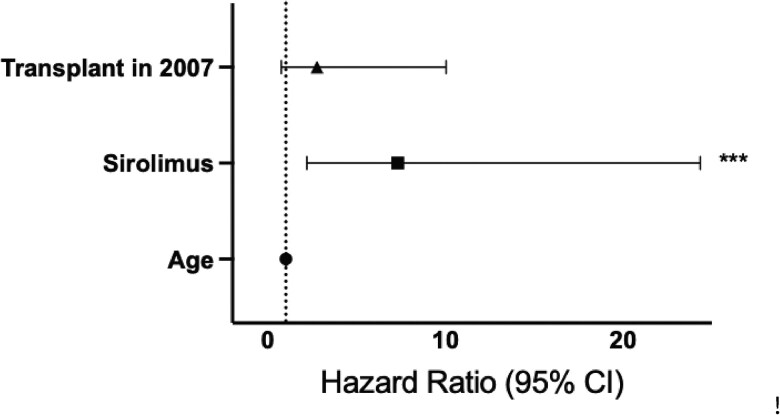
Multivariate analysis of clinical variables and their associated risk of RSV infection. A multivariate Cox regression analysis was performed on clinical variables that had a *P* value ≤ 0.2 in univariate analysis. Six clinical variables met this criterion: age, transplant year in 2007, use of sirolimus as part of GVHD prophylaxis, having an ANC < 1000, having an ALC < 100, and having an AMC < 100. To avoid over-adjustment, cell counts were excluded given their association with hospitalization post-transplant and therefore reduced exposure to respiratory viruses in the community. The dotted line is a hazard ratio of one, which represents a null association. The asterisks indicate *P* = 0.0012.

Using only variables with a *P* value ≤ 0.2 from our univariate analysis (Table 1), we then performed a multivariate Cox regression analysis (Figure 2) that included recipient age, transplant year in 2007, and use of sirolimus as part of graft-versus-host disease (GVHD) prophylaxis. Low cell counts were excluded from the multivariate analysis given their association with hospitalization post-transplant and concomitant reduced exposure to respiratory viruses in the community. After adjusting for age and transplant year, using sirolimus for GVHD prophylaxis was associated with an increased risk of RSV infection in the multivariate analysis (HR: 7.3, 95% CI: 2.2–24.3). Since RSV incidence in the community can vary year-to-year, we examined the proportion of participants receiving sirolimus and the proportion of participants with RSV detected in the first 100 days post-transplant by calendar year (Supplementary Table S5). The proportion of patients receiving sirolimus was relatively similar across all years (average 4.8%, range 2.4%–9.1%). The average proportion of patients with RSV similarly was 4.7% (range 2.4%–8.8%) between 2006 and 2008. However, there were no cases of RSV in 2010 due to low enrollment (*N* = 11 out of a total of 471). Overall, the frequency of RSV detection and sirolimus administration was low, leading to a wide confidence interval for sirolimus in the multivariate analysis.

### Serum Neutralizing Antibody and Risk for RSV Infection Post-Transplant

**Figure 3.**
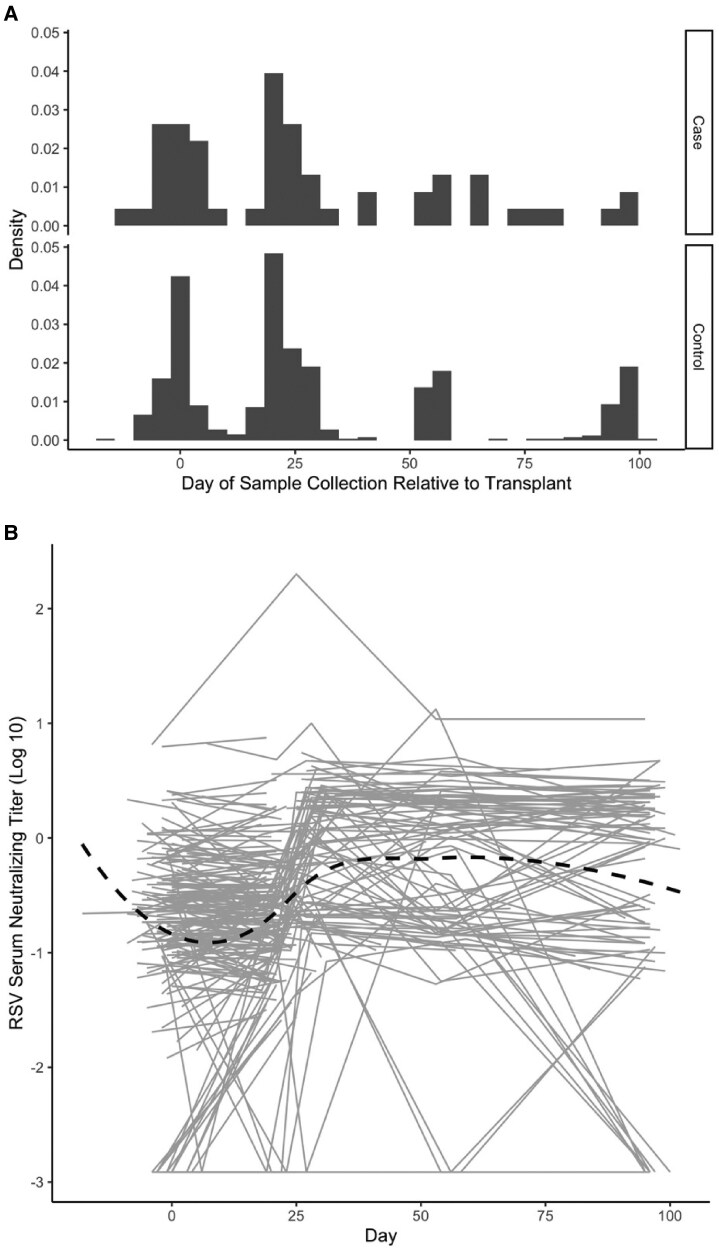
Analysis of serum neutralizing antibody titers to RSV post-transplant. *A,* Distribution of serum samples collected by day relative to transplant for cases and controls. Density on the y-axis indicates the proportion of total samples analyzed. *B*, Serum neutralizing antibody titers to RSV relative to day of transplant in control participants. Each gray line represents results from a control participant. The dotted line represents the overall trend for all control participants analyzed.

Participants in the analysis had serum samples collected from 2 to 6 time points, from 1 week before their transplant to 100 days after transplant (Figure 3a; Supplementary Figure S1 and Table S6). Of the 16 participants with RSV detected within the first 100 days post-transplant, serum neutralizing antibody titers were available for 8 individuals post-infection (Supplementary Figure S2). After infection, 6 out of the 8 (75%) failed to mount at least a 0.5 log_10_ rise in serum RSV neutralizing antibody titer, whereas the other 2 (25%) mounted at least a 1 log_10_ rise in serum RSV neutralizing antibody titer compared with pre-transplant/peri-transplant antibody titers. All 6 that failed to mount a significant response had RSV detection within the first 28 days post-transplant, whereas the 2 that demonstrated a significant rise in serum neutralizing RSV antibody titer experienced RSV infection beyond 28 days post-transplant. Among control participants without RSV infection within the first 100 days post-transplant, serum neutralizing antibody titers exhibited a biphasic pattern, characterized by an initial rapid decline, followed by a recovery phase, and then a slower secondary decline (Figure 3b).

**Figure 4.**
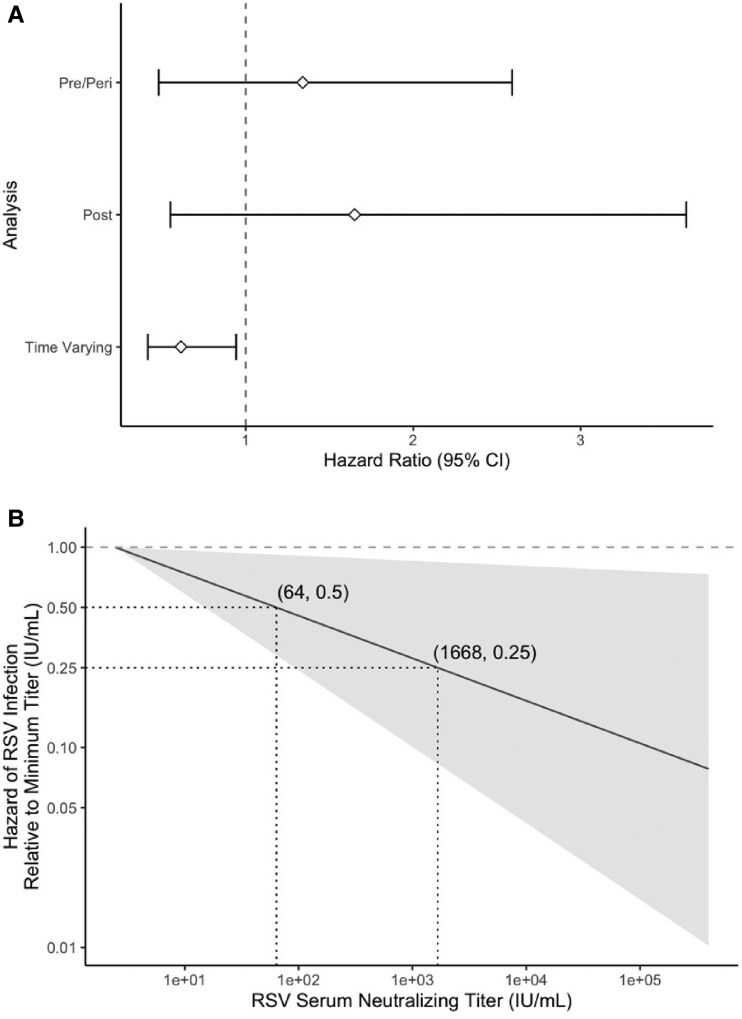
Correlation between serum neutralizing antibody titers and risk for RSV infection. *A*, Hazard ratios for PCR-confirmed RSV infection in relation to serum neutralizing antibody levels. Hazard ratios were calculated using the Cox proportional hazards model. Circles represent the hazard ratio, and the horizontal bars represent the range from the lower to the upper limit of the 95% confidence interval. The dotted line is a hazard ratio of 1, which represents a null association. In the time-varying analysis, the most recent serum neutralizing antibody titer was found to be significantly associated with instantaneous risk of PCR-detected RSV infection at a given time (HR: 0.61, 95% CI: 0.41–0.96). *B*, Estimated hazard of PCR-detected RSV infection based on the most recent RSV serum neutralizing titer. Hazards are relative to the minimum observed serum neutralizing titer (IU/mL) in the cohort. The most recent RSV serum neutralizing titer is from serum collected within 28 days of PCR detection of RSV. Estimates were calculated using the time-varying hazard ratio from the Cox proportional hazard model. The implemented Cox model specifies a linear relationship between the log relative hazard and the log titer. Relative hazards based on the 95% confidence intervals (calculated via bootstrap) are shown shaded in gray. A relative hazard of 1, indicating the same hazard of RSV infection as those with the minimum serum neutralizing titer, is shown via a dotted line. The minimum RSV serum neutralizing titer was 2.44 IU/mL, and the maximum titer was 4 × 10^5^ IU/mL. Distances on both the x- and y-axes are on a log_10_ scale.

Hazard ratios were used to determine if the log transformed levels of antibody measured in serum samples were associated with time to detected RSV infection (Figure 4a). Due to the removal of participants infected before the second time point in the post-transplant analysis and censoring of participants with antibody measures older than 28 days in the time varying analysis, event counts varied slightly by analysis (Supplementary Table S7). Neither pre-transplant/peri-transplant or post-transplant serum neutralizing antibody levels were significantly associated with risk of PCR-detected RSV infection (Figure 4a; Supplementary Table S8). As the pre-transplant/peri-transplant or post-transplant analysis relied on antibody levels collected at time points that could be relatively distant from the time of infection, correlations, if any, between these antibody levels and risk for RSV infection would be weak. Therefore, we also performed a time-varying analysis in which antibody levels closer to the time of infection were used. Among those not yet infected at a given time, participants with a 10-fold higher most recent serum neutralizing antibody titer had a significantly lower instantaneous risk of detected RSV infection than those with a 10-fold lower antibody level at that time (HR: 0.61, 95% CI: 0.42-0.94) (Figure 4a). The estimated risk of RSV infection significantly decreased with increasing serum neutralizing antibody titer (Figure 4b). For example, a titer of 64 IU/mL was associated with 0.5 times the hazard of infection, and a titer of 1,668 IU/mL was associated with 0.25 times the hazard of infection, compared with the lowest observed serum RSV neutralizing antibody titer in the study (2.44 IU/mL). These findings indicate that neutralizing antibody titers in serum samples are correlated with a patient's risk of RSV infection after HCT.

## DISCUSSION

Correlates of protection have largely been understudied in immunocompromised populations [30–32]. In this study, we followed 471 allogeneic HCT recipients longitudinally with weekly nasal wash PCRs for respiratory viral detection, blood draws, and symptom surveys. We observed RSV infection in 3.4% of participants within the first 100 days post-transplant, a period of heightened vulnerability due to a substantially immunocompromised state [2–7]. Three out of 16 RSV cases (19%) showed evidence of lower respiratory tract involvement, consistent with prior reports estimating that 19%-36% of RSV infections involve the lower respiratory tract [33, 34]. We found that the risk for RSV infection was significantly increased when sirolimus was used as prophylaxis for GVHD. Importantly, we also found that lower levels of RSV neutralizing antibody from serum samples collected prior to infection were significantly correlated with increased risk of RSV infection in the first 100-days post-transplant. Taken together, these findings provide the first evidence that immunologic predictors and serum antibody levels can be leveraged to identify at-risk HCT recipients and to guide the development of strategies and the design of clinical trials for RSV prevention in this vulnerable patient population.

Several studies have examined correlates of protection to RSV after vaccination or mAb administration in nonimmunocompromised patients [13, 16–18, 35, 36]. These studies have provided valuable information on correlates of protection from severe RSV disease with vaccine-induced serum neutralizing and anti-F antibodies in infants and older adults [37]. However, less is known about the risk factors and correlates of protection for HCT recipients. In a previous retrospective study, we enrolled 181 HCT recipients with RSV infection of the URT to determine if higher neutralizing antibody levels were correlated with protection from viral progression to the LRT [9]. We did not observe a significant correlation between risk of RSV progression with neutralizing antibody levels just prior to transplant or neutralizing antibody levels near the time of RSV detection in the URT. Therefore, the importance of serum RSV antibody levels in protecting HCT recipients from RSV disease remained unclear. The present study, leveraging a time varying analysis of a prospective, longitudinal cohort of HCT recipients, is the first to demonstrate a significant correlation between lower levels of serum neutralizing antibodies and risk of RSV infection in HCT recipients. The increased risk likely is a result of waning recipient serum antibodies over time. In the early post-transplant period, HCT recipients have incomplete humoral immune reconstitution. As recipient serum antibodies wane with time after HCT, the risk for respiratory viral infections increases [5–7, 38]. IVIG is occasionally used to boost immunoglobulin levels after HCT, and some studies have shown variable benefit in boosting antibody titers to RSV after HCT [39]. While only 3 participants in the present cohort received IVIG within the first 100 days post-transplant, none of the 3 tested positive for RSV.

In this cohort, we found that most participants with RSV infection in the first 100 days failed to mount a significant serum neutralizing antibody response to RSV after infection. Those that failed had RSV detected within the first month of transplant. The 2 participants who experienced a significant rise in serum RSV neutralizing antibody titer had RSV infection beyond the first month of transplant. Although the sample size is small, these results suggest that some HCT recipients can mount a strong humoral immune response to RSV infection, and possibly even RSV vaccination, depending on the time of exposure relative to transplant. Of note, half of the cases in our cohort had RSV detected within the first month of transplant, when vaccination is likely ineffective. We also observed a rapid decline in serum neutralizing antibody titers to RSV early post-transplant. Therefore, prevention of these early infections would require pre-transplant prophylaxis. However, since half of cases had RSV detected later in transplant, durable protection beyond 30 days is also important.

Limitations of this study include the small sample size, which led to relatively low precision in estimating the effects of covariates. Due to the sample size, we did not attempt to account for confounding in our time-varying analysis of serum neutralizing antibody levels. Another potential limitation is the generalizability of results from HCT recipients in 2005-2010 to HCT recipients now. Clinical risk factors, including conditioning regimens and GHVD prophylaxis regimens have evolved over time. However, to our knowledge, this is the only longitudinal, prospective cohort of HCT recipients who were followed with weekly nasal swabs for respiratory viral diagnostic testing. This resource-intensive study design was necessary to enable a time-varying analysis of serum neutralizing antibody levels. Although costly to reproduce, another more contemporary multicenter study with a larger sample size will be needed to validate our results. The impact of serum neutralizing antibodies on risk for RSV infection, however, is less likely to be influenced by when the study was performed. Another limitation is that antibody assays for cases were performed separately from controls. However, we utilized the RSV WHO standard in each assay to correct for batch effects, as well as make our results comparable to other clinical studies where this standard was utilized. Additionally, the diagnostic PCR used clinically in this study did not distinguish between RSV subtypes A and B. Although neutralization assays were performed with just RSV subtype A, there is a positive correlation between serum neutralizing titers to RSV-A and RSV-B due to the similarity between the viruses leading to serologic cross-reactivity [40]. A strong association between RSV-A neutralizing activity with protection against all RSV infections has also been previously demonstrated [41, 42]. Another limitation of our time to event analyses is the assumption that loss to follow-up was unrelated to time to infection. Our Cox regression analyses also assume proportional hazards, which are constant over time and a linear relationship (or log linear relationship for antibody measures) between predictors and the log hazard of PCR-detected RSV infection. Due to the small sample size, however, diagnostic plots (Supplementary Figure S3) assessing the proportionality of hazards and fit of the linear model for the humoral analyses could not definitively conclude whether these assumptions were valid. Other vaccine studies have similarly utilized Cox regression analysis to analyze antibody levels as a correlate of risk for RSV or SARS-CoV-2 infection [43, 44]. Overall, a future study with larger sample size will be needed to validate the robustness of these results.

In our analysis of clinical covariates, we observed an increased risk of RSV infection in participants receiving sirolimus for GVHD prophylaxis compared with participants not receiving sirolimus. The increased risk of RSV infection contrasts with the effect observed with CMV, in which mTOR inhibition is protective against CMV reactivation and replication [45]. This is likely a result of differences in the pathogenesis of CMV—a herpes virus primarily controlled by T cell immunity—compared with respiratory viruses, in which innate immunity and neutralizing antibodies block infection at mucosal sites of entry. For example, mTOR inhibition exerts a dose dependent immunostimulatory effect on CMV-specific CD8^+^ T cells that suppress CMV reactivation [45]. In contrast, inhibition of the mTOR pathway with physiologic levels of sirolimus can lead to significantly increased RSV protein production and viral replication in vitro [46]. This phenomenon has also been observed with other respiratory virus, including influenza, in mice treated with sirolimus [47]. The mechanism by which sirolimus affects RSV replication may be related to suppression of innate immunity that would otherwise clear virus [48]. Notably, overall immunosuppression is likely associated with increased risk of RSV infection, but we could not estimate hazard ratios for calcineurin inhibitors or post-transplant cyclophosphamide because all patients with RSV received a calcineurin inhibitor and none received post-transplant cyclophosphamide. A trend toward increased risk for RSV detection was observed with steroid usage, but this was not statistically significant, due to the large proportion of participants in the control group that were also on steroids. Larger prospective multicenter studies are needed to validate these results. Sirolimus and post-transplant cyclophosphamide use for GVHD prophylaxis was relatively infrequent during the years of this study (2005-2010), and these agents were used in combination with other immunosuppressive drugs. Although statistically significant, the association between RSV infection and sirolimus should also be interpreted in the context of the small sample size. Thus, the impact of these medications on RSV infection will need to be validated in more recent cohorts and with more power, as the use of these agents for GVHD prophylaxis is growing.

Serum neutralizing antibody levels have been identified as a correlate of protection in numerous RSV vaccine and mAb studies [49]. However, these studies have primarily focused on nonimmunocompromised patients, and generalizability to vulnerable populations, including HCT recipients, is understudied. The demonstration of serum neutralizing antibody levels as an inverse correlate of risk in this present study has implications for enabling clinical trials and immunobridging studies of vaccines and mAbs in HCT recipients and other immunocompromised populations. Inferred efficacy through immunobridging has already been used successfully to make RSV vaccination available to younger immunocompromised patients [50]. The design of future clinical trials and immunobridging studies could leverage serum RSV neutralizing antibody levels to develop strategies aimed at preventing poor outcomes from RSV infection in vulnerable, immunocompromised patients.

## Supplementary Material

ofag005_Supplementary_Data

## Supplementary Data

Supplementary materials are available at *Open Forum Infectious Diseases* online. Consisting of data provided by the authors to benefit the reader, the posted materials are not copyedited and are the sole responsibility of the authors, so questions or comments should be addressed to the corresponding author.
