# Supplementary material for: Respiratory Syncytial Virus in Hematopoietic Cell Transplant Recipients: Clinical and Humoral Risk Factors for Infection
Source: Open Forum Infect Dis. 2026 Jan 20;13(1):ofag005. doi: 10.1093/ofid/ofag005 (PMC12828088; doi:10.1093/ofid/ofag005)
Supplement: ofag005_Supplementary_Data [file ofag005_supplementary_data.docx]

**SUPPLEMENTARY FIGURES**


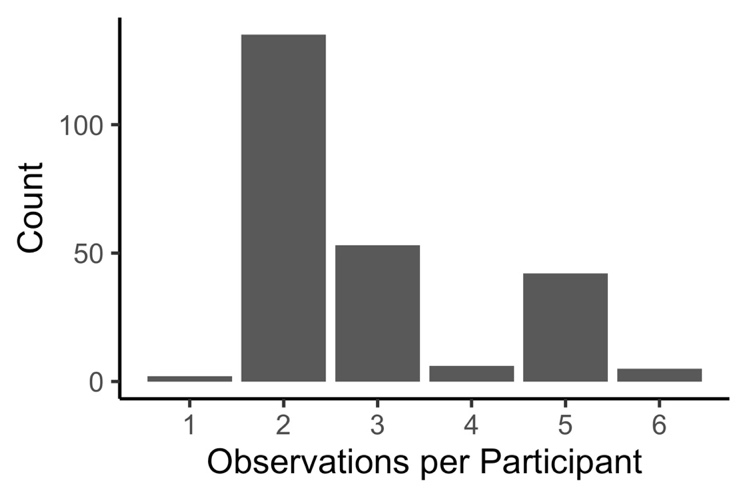


**Figure S1. Number of serum samples analyzed per participant.** Samples for which a titer could not be calculated were excluded from these counts. Of the 241 subjects with serum samples analyzed, 210 (87%) subjects had pre-/peri- transplant measurements.


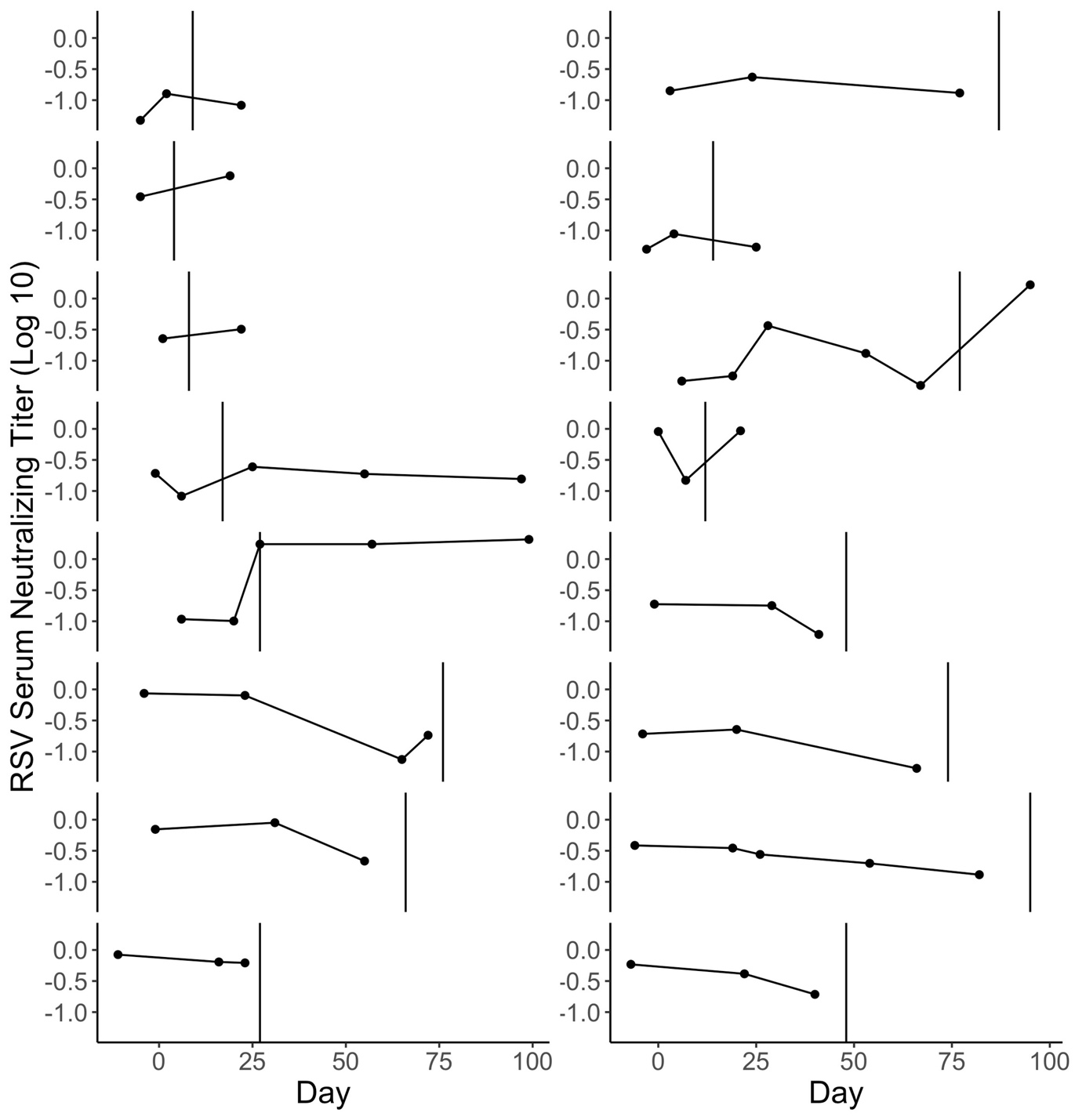


**Figure S2. Line plots of log_10_ RSV serum neutralizing titer by days relative to transplant (day 0) for participants who had a positive RSV PCR within 100 days of transplant**. N=16 with 8 participants shown on the left and 8 shown on the right. Each dot is a neutralizing titer measurement. The vertical lines indicate the day of a positive RSV PCR result for that individual.


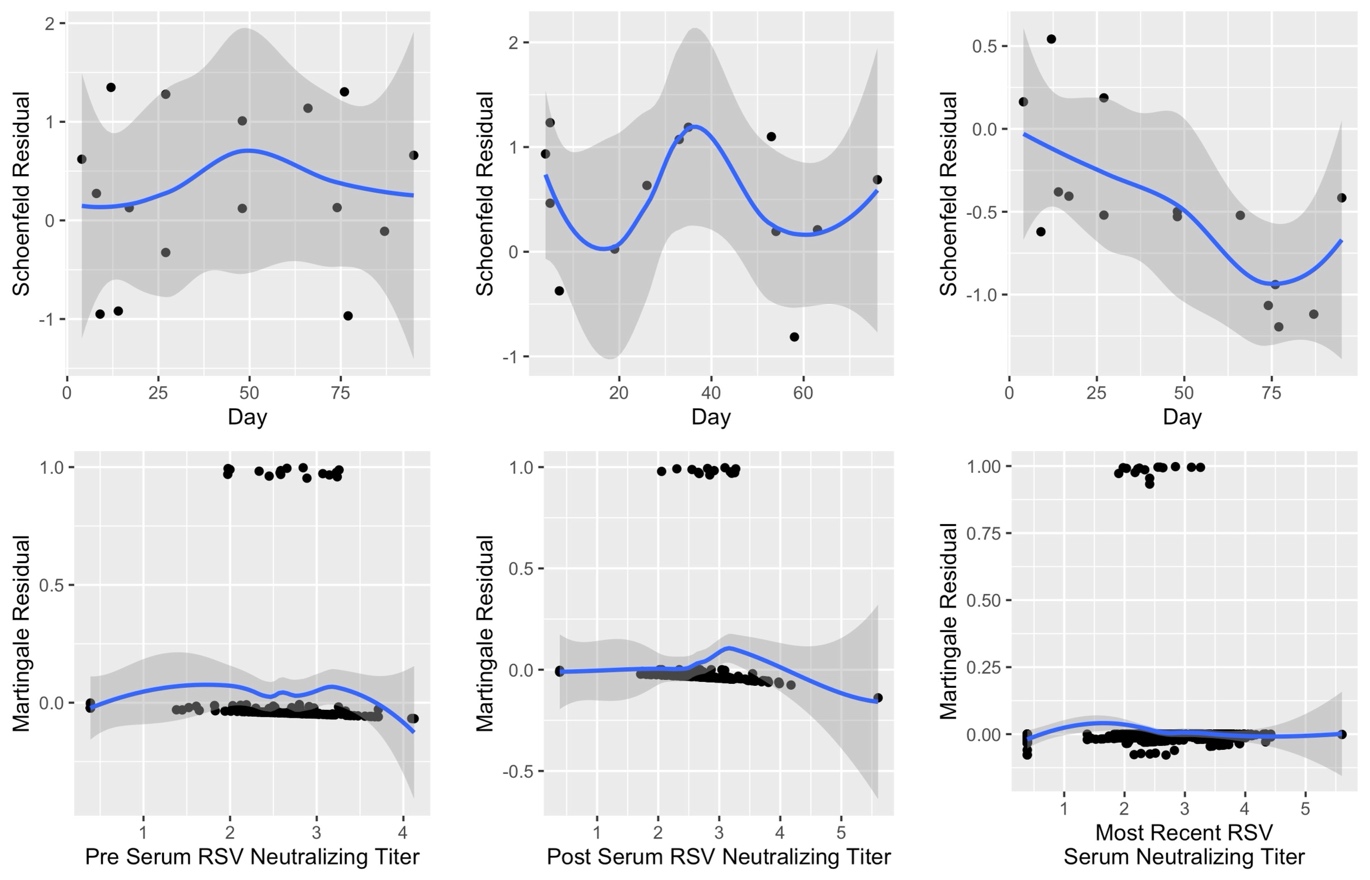


**Figure S3.** **Schoenfeld and Martingale residual plots for the serum neutralizing titer analyses.** The left column shows the pre-/peri-transplant timepoint, the middle column shows the post-transplant timepoint, and the right column shows the time varying analysis. The serum neutralizing titer is in log_10_ IU/mL.

**SUPPLEMENTARY TABLES**

**Table S1. Baseline clinical variables of sampled controls, unsampled controls, and the overall control cohort**

|  | **In serum sample analysis (N=225)** | **Not in serum sample analysis (N=230)** | **Overall controls (N=455)** |
| --- | --- | --- | --- |
| **Days follow-up** |  |  |  |
| Mean (SD) | 241 (131) | 188 (137) | 214 (137) |
| Median [Q1, Q3] | 270 [7.00, 400] | 106 [1.00, 396] | 184 [1.00, 400] |
| Missing | 1 (0.4%) | 2 (0.9%) | 3 (0.7%) |
| **Recipient age** |  |  |  |
| Mean (SD) | 47.0 (17.4) | 45.1 (18.6) | 46.0 (18.1) |
| Median [Q1, Q3] | 50.9 [0.819, 75.2] | 49.8 [0.739, 74.9] | 50.4 [0.739, 75.2] |
| **Recipient age 60 and older** |  |  |  |
| Mean (SD) | 0.276 (0.448) | 0.230 (0.422) | 0.253 (0.435) |
| Median [Q1, Q3] | 0 [0, 1.00] | 0 [0, 1.00] | 0 [0, 1.00] |
| **Recipient age 10 and under** |  |  |  |
| Mean (SD) | 0.0444 (0.207) | 0.0609 (0.240) | 0.0527 (0.224) |
| Median [Q1, Q3] | 0 [0, 1.00] | 0 [0, 1.00] | 0 [0, 1.00] |
| **Recipient sex** |  |  |  |
| Female | 89 (39.6%) | 77 (33.5%) | 166 (36.5%) |
| Male | 136 (60.4%) | 153 (66.5%) | 289 (63.5%) |
| **Diagnosis** |  |  |  |
| ALL | 23 (10.2%) | 33 (14.3%) | 56 (12.3%) |
| AML | 70 (31.1%) | 89 (38.7%) | 159 (34.9%) |
| CLL | 15 (6.7%) | 9 (3.9%) | 24 (5.3%) |
| CML | 10 (4.4%) | 9 (3.9%) | 19 (4.2%) |
| Lymphomas | 27 (12.0%) | 22 (9.6%) | 49 (10.8%) |
| MDS | 28 (12.4%) | 24 (10.4%) | 52 (11.4%) |
| Myeloma | 16 (7.1%) | 16 (7.0%) | 32 (7.0%) |
| Other diseases | 36 (16.0%) | 28 (12.2%) | 64 (14.1%) |
| **Smoking status** |  |  |  |
| Never | 140 (62.2%) | 141 (61.3%) | 281 (61.8%) |
| Current | 15 (6.7%) | 15 (6.5%) | 30 (6.6%) |
| Former | 62 (27.6%) | 63 (27.4%) | 125 (27.5%) |
| Unknown | 8 (3.6%) | 11 (4.8%) | 19 (4.2%) |
| **Transplant type** |  |  |  |
| Non-Myeloablative | 105 (46.7%) | 84 (36.5%) | 189 (41.5%) |
| Myeloablative+no TBI | 65 (28.9%) | 75 (32.6%) | 140 (30.8%) |
| Myeloablative+TBI | 55 (24.4%) | 71 (30.9%) | 126 (27.7%) |
| **Exposure to children** |  |  |  |
| No | 153 (68.0%) | 150 (65.2%) | 303 (66.6%) |
| Yes | 67 (29.8%) | 72 (31.3%) | 139 (30.5%) |
| Missing | 5 (2.2%) | 8 (3.5%) | 13 (2.9%) |
| **Exposure to children under 4** |  |  |  |
| No | 181 (80.4%) | 187 (81.3%) | 368 (80.9%) |
| Yes | 44 (19.6%) | 43 (18.7%) | 87 (19.1%) |
| **Season of transplant** |  |  |  |
| fall | 31 (13.8%) | 66 (28.7%) | 97 (21.3%) |
| spring | 53 (23.6%) | 48 (20.9%) | 101 (22.2%) |
| summer | 75 (33.3%) | 56 (24.3%) | 131 (28.8%) |
| winter | 66 (29.3%) | 60 (26.1%) | 126 (27.7%) |
| **Graft type** |  |  |  |
| REL/MATCHED | 67 (29.8%) | 83 (36.1%) | 150 (33.0%) |
| REL/HAPLOIDENTICAL | 9 (4.0%) | 13 (5.7%) | 22 (4.8%) |
| REL/MISMATCH | 4 (1.8%) | 3 (1.3%) | 7 (1.5%) |
| URD/CORD | 15 (6.7%) | 30 (13.0%) | 45 (9.9%) |
| URD/MATCHED | 116 (51.6%) | 89 (38.7%) | 205 (45.1%) |
| URD/MISMATCH | 14 (6.2%) | 12 (5.2%) | 26 (5.7%) |
| **Graft source** |  |  |  |
| Bone marrow | 37 (16.4%) | 44 (19.1%) | 81 (17.8%) |
| Cord blood | 15 (6.7%) | 30 (13.0%) | 45 (9.9%) |
| Peripheral blood | 173 (76.9%) | 156 (67.8%) | 329 (72.3%) |
| **Post-transplant cyclophosphamide** |  |  |  |
| FALSE | 216 (96.0%) | 214 (93.0%) | 430 (94.5%) |
| TRUE | 9 (4.0%) | 16 (7.0%) | 25 (5.5%) |
| **Rapamycin** |  |  |  |
| FALSE | 219 (97.3%) | 222 (96.5%) | 441 (96.9%) |
| TRUE | 6 (2.7%) | 8 (3.5%) | 14 (3.1%) |
| **Calcineurin inhibitors** |  |  |  |
| FALSE | 1 (0.4%) | 3 (1.3%) | 4 (0.9%) |
| TRUE | 224 (99.6%) | 227 (98.7%) | 451 (99.1%) |
| **MMF** |  |  |  |
| FALSE | 101 (44.9%) | 109 (47.4%) | 210 (46.2%) |
| TRUE | 124 (55.1%) | 121 (52.6%) | 245 (53.8%) |
| **MTX** |  |  |  |
| FALSE | 122 (54.2%) | 129 (56.1%) | 251 (55.2%) |
| TRUE | 103 (45.8%) | 101 (43.9%) | 204 (44.8%) |
| **CMV status** |  |  |  |
| D-/R- | 73 (32.4%) | 78 (33.9%) | 151 (33.2%) |
| D-/R+ | 87 (38.7%) | 73 (31.7%) | 160 (35.2%) |
| D+/R- | 18 (8.0%) | 22 (9.6%) | 40 (8.8%) |
| D+/R+ | 47 (20.9%) | 57 (24.8%) | 104 (22.9%) |

**Table S2. Samples with antibody values outside the range of the standard curve**. The number and corresponding percentage of samples with values below or above the WHO standard are provided. Counts and totals are of the samples used in analyses.

| **Assay** | **Count Over the Limit of Detection (%)** | **Count Under the Limit of Detection (%)** |
| --- | --- | --- |
| Serum Neutralizing Titer | 2 (0%) | 37 (5%) |

**Table S3.** Summary of exploratory analyses not presented in this paper, with rationale for exclusion and statistical significance finding. The outcomes considered were first detected infection with RSV, first detected infection with HMPV, first detected infection with HPIV3, or first detected infection with any of the 3 (denoted as “All”). The measures considered were virus-specific nasal neutralizing antibody titer, virus-specific nasal IgA, and virus-specific serum neutralizing antibody titer. For the analyses of first detection with any of the 3 viruses (“All” outcome), an average of the virus-specific measures on the log_10_ scale was used. The time points considered were pre/peri-transplant, post-transplant, and time-varying. Of the 33 analyses aside from those presented, 9 were judged unreliable due to a low event count, one was significant, and 23 were not significant at a 0.05 significance threshold. Note that these analyses were conducted on preliminary PCR results with minor differences in analysis methods.

| **Measure** | **Time Point** | **Outcome** | **Hazard Ratio** | **95% CI** | **p value** | **Rationale for Exclusion** |
| --- | --- | --- | --- | --- | --- | --- |
| Nasal Neutralizing | Pre/Peri | RSV | 1.29 | 0.07-3.21 | 0.80 | Nasal measures unreliable due to lack of normalization for dilution. |
| Nasal IgA | Pre/Peri | RSV | 0.69 | 0.33-1.55 | 0.34 |  |
| Nasal Neutralizing | Post | RSV | 3.09 | 0.40-13.67 | 0.27 |  |
| Nasal IgA | Post | RSV | 0.65 | 0.30-1.53 | 0.71 |  |
| Nasal Neutralizing | Time-varying | RSV | 0.79 | 0.35-1.37 | 0.50 |  |
| Nasal IgA | Time-varying | RSV | 0.86 | 0.63-1.16 | 0.33 |  |
| Serum Neutralizing | Pre/Peri | All | 0.77 | 0.33-1.69 | 0.54 | Combined virus outcome limited by low likelihood for antibodies against one virus to neutralize another virus (cross-neutralization) |
| Nasal Neutralizing | Pre/Peri | All | 3.68 | 1.30-8.51 | 0.01 |  |
| Nasal IgA | Pre/Peri | All | 0.90 | 0.69-1.16 | 0.42 |  |
| Serum Neutralizing | Post | All | 1.23 | 0.32-2.92 | 0.71 |  |
| Nasal Neutralizing | Post | All | 2.25 | 0.74-5.20 | 0.11 |  |
| Nasal IgA | Post | All | 0.81 | 0.53-1.15 | 0.29 |  |
| Serum Neutralizing | Time-varying | All | 0.66 | 0.40-1.13 | 0.13 |  |
| Nasal Neutralizing | Time-varying | All | 0.99 | 0.49-1.79 | 0.98 |  |
| Nasal IgA | Time-varying | All | 1.03 | 0.76-1.30 | 0.85 |  |
| Serum Neutralizing | Pre/Peri | HMPV | 1.38 | 0.26-3.33 | 0.62 | Results unreliable due to low event count (N=10) |
| Nasal Neutralizing | Pre/Peri | HMPV | 1.19 | 0.35-2.10 | 0.70 |  |
| Nasal IgA | Pre/Peri | HMPV | 1.03 | 0.65-1.34 | 0.89 |  |
| Serum Neutralizing | Post | HMPV | 0.86 | 0.51-1.30 | 0.51 |  |
| Nasal Neutralizing | Post | HMPV | 0.76 | 0.40-1.22 | 0.34 |  |
| Nasal IgA | Post | HMPV | 0.89 | 0.80-1.04 | 0.11 |  |
| Serum Neutralizing | Time-varying | HMPV | 1.14 | 0.27-2.28 | 0.92 |  |
| Nasal Neutralizing | Time-varying | HMPV | 0.87 | 0.46-1.39 | 0.63 |  |
| Nasal IgA | Time-varying | HMPV | 1.30 | 0.92-1.65 | NA |  |
| Serum Neutralizing | Pre/Peri | HPIV3 | 1.00 | 0.30-2.57 | 1.00 | Exploratory due to lower priority and lower event count (N=14) than RSV |
| Nasal Neutralizing | Pre/Peri | HPIV3 | 1.07 | 0.43-1.96 | 0.91 |  |
| Nasal IgA | Pre/Peri | HPIV3 | 0.87 | 0.55-1.21 | 0.50 |  |
| Serum Neutralizing | Post | HPIV3 | 0.95 | 0.28-1.79 | 0.91 |  |
| Nasal Neutralizing | Post | HPIV3 | 0.78 | 0.19-1.60 | 0.92 |  |
| Nasal IgA | Post | HPIV3 | 0.67 | 0.16-1.19 | 0.60 |  |
| Serum Neutralizing | Time-varying | HPIV3 | 1.06 | 0.10-2.35 | 0.94 |  |
| Nasal Neutralizing | Time-varying | HPIV3 | 1.05 | 0.39-1.97 | 0.91 |  |
| Nasal IgA | Time-varying | HPIV3 | 0.85 | 0.40-1.25 | 0.57 |  |

**Table S4. Clinical outcomes of participants with RSV infection.**

| Variable | Total, n= 471 (%) | Control, n=455 (%) | RSV, n= 16 (%) |
| --- | --- | --- | --- |
| Death Before Day 100* | 43 (9) | 42 (9) | 1 (6) |
| RSV outcomes |  |  |  |
| LRTI** | NA | NA | 3 (19) |
| Received ribavirin | NA | NA | 4 (25) |
| Required Hospitalization*** | NA | NA | 4 (25) |

* No deaths were attributable to RSV infection

** Lower respiratory tract infection was determined by chest X-ray.

*** Hospitalization occurred if patients were readmitted to hospital due to RSV infection or if they were already admitted but required care that would have caused admission had they been outpatient at time of infection.

Abbreviations: LRTI, lower respiratory tract infection; NA, not applicable.

**Table S5. Proportion of participants on sirolimus for GVHD prophylaxis and proportion of participants with RSV detected within the first 100 days post-transplant by calendar year.**

| Year | Sirolimus (%) | RSV detection in the first 100 days post-transplant (%) | Number of participants |
| --- | --- | --- | --- |
| 2006 | 4.0 | 3.0 | 99 |
| 2007 | 5.3 | 8.8 | 114 |
| 2008 | 2.4 | 2.4 | 126 |
| 2009 | 3.3 | 0 | 121 |
| 2010 | 9.1 | 0 | 11 |

**Table S6. Distribution of samples collected by day relative to transplant for cases and controls.**

|  | Case (N=56 samples) | Control (N=631 samples) | Overall (N=687 samples) |
| --- | --- | --- | --- |
| Day |  |  |  |
| Mean (SD) | 27.2 (29.4) | 28.9 (31.2) | 28.7 (31.0) |
| Median [Q1, Q3] | 22.0 [-11.0, 99.0] | 21.0 [-18.0, 100] | 21.0 [-18.0, 100] |

**Table S7. Event counts by analysis.** Event counts varied slightly by analysis due to differences in which samples were chosen, the removal of participants infected before the second time-point in the post analysis, and the censoring of participants with antibody measures older than 28 days in the time varying analysis.

| **Assay** | **Pre/Peri Transplant** | **Post-Transplant** | **Time Varying** |
| --- | --- | --- | --- |
| Serum Neutralizing Titer | 16 | 13 | 15 |

**Table S8. Median antibody values.** Summary data were calculated after imputing values for out-of-range observations. Values in parentheses are the interquartile range. Cases and controls are defined according to the event definitions for the pre-/peri- and post-transplant timepoints, as described in the Statistical Analysis section. Median and interquartile range for time varying observations were calculated across all observations (multiple per participant).

|  | | **Serum Neutralizing Titer** | **Titer Normalized to WHO** |
| --- | --- | --- | --- |
| Pre/Peri Transplant | All | 0.194 (0.106-0.448) | 388 (212-976) |
|  | Cases | 0.209 (0.133-0.615) | 418 (266-1230) |
|  | Controls | 0.194 (0.105-0.433) | 388 (210-866) |
| Post Transplant | All | 0.228 (0.110-0.430) | 456 (220-860) |
|  | Cases | 0.350 (0.227-0.757) | 700 (218-1514) |
|  | Controls | 0.211 (0.109-0.392) | 422 (422-784) |
